# The STAT1/HMGB1/NF-κB pathway in chronic inflammation and kidney injury after cisplatin exposure

**DOI:** 10.7150/thno.81406

**Published:** 2023-05-08

**Authors:** Ying Fu, Yu Xiang, Ying Wang, Zhiwen Liu, Danyi Yang, Jie Zha, Chengyuan Tang, Juan Cai, Guochun Chen, Zheng Dong

**Affiliations:** 1 Department of Nephrology, Hunan Key Laboratory of Kidney Disease and Blood Purification, The Second Xiangya Hospital of Central South University, Changsha 410011, China.; 2 Department of Cellular Biology and Anatomy, Medical College of Georgia at Augusta University and Charlie Norwood VA Medical Center, Augusta, GA 30912, USA.

**Keywords:** cisplatin, inflammation, renal fibrosis, HMGB1, NF-κB

## Abstract

**Rationale:** Cisplatin, a potent chemotherapeutic drug, induces side effects in normal tissues including the kidney. To reduce the side effects, repeated low-dose cisplatin (RLDC) is commonly used in clinical setting. While RLDC reduces acute nephrotoxicity to certain extents, a significant portion of patients later develop chronic kidney problems, underscoring the need for novel therapeutics to alleviate the long-term sequelae of RLDC therapy.

**Methods:** *In vivo*, the role of HMGB1 was examined by testing HMGB1 neutralizing antibodies in RLDC mice. *In vitro*, the effects of HMGB1 knockdown on RLDC-induced nuclear factor-κB (NF-κB) activation and fibrotic phenotype changes were tested in proximal tubular cells. To study signal transducer and activator of transcription 1 (STAT1), siRNA knockdown and its pharmacological inhibitor Fludarabine were used. We also searched the Gene Expression Omnibus (GEO) database for transcriptional expression profiles and evaluated kidney biopsy samples from CKD patients to verify the STAT1/HMGB1/NF-κB signaling axis.

**Results:** We found that RLDC induced kidney tubule damage, interstitial inflammation, and fibrosis in mice, accompanied by up-regulation of HMGB1. Blockage of HMGB1with neutralizing antibodies and Glycyrrhizin suppressed NF-κB activation and associated production of pro-inflammatory cytokines, reduced tubular injury and renal fibrosis, and improved renal function after RLDC treatment. Consistently, knockdown of HMGB1 decreased NF-κB activation and prevented the fibrotic phenotype in RLDC-treated renal tubular cells. At the upstream, knockdown of STAT1 suppressed HMGB1 transcription and cytoplasmic accumulation in renal tubular cells, suggesting a critical role of STAT1 in HMGB1 activation. Upregulation of STAT1/HMGB1/NF-κB along with inflammatory cytokines was also verified in kidney tissues of CKD patients.

**Conclusion:** These results unravel the STAT1/HMGB1/NF-κB pathway that contributes to persistent inflammation and chronic kidney problems after cisplatin nephrotoxicity, suggesting new therapeutic targets for kidney protection in cancer patients receiving cisplatin chemotherapy.

## Introduction

Nephrotoxicity is the main limiting factor for the clinical use and efficacy of cisplatin, which causes acute kidney injury (AKI) in almost 30% of patients 1, 2. Even with measures to reduce acute nephrotoxicity, such as treatment regimen of repeated low-dose cisplatin (RLDC) 3, patients after cisplatin exposure are still at risk of progressive decline of the renal function and development of chronic kidney disease (CKD) in the long-term 4, 5. To study the chronic effects of cisplatin nephrotoxicity, several animal models of RLDC treatment were established recently 6-12. In addition, we have developed a cell model that mimics RLDC treatment *in vivo* 8 and reproduces the pro-fibrotic phenotype in a proximal tubule cell line after four cycles of low-dose cisplatin treatment. These models provide the tools for studying the pathogenesis of chronic kidney problems after cisplatin exposure.

Persistent inflammation is an important factor in the development of chronic kidney problems and CKD 13. Renal inflammation involves immune cells infiltrating the kidney and the residual kidney cells, such as the renal tubular epithelial cells 14. These cells and other elements including some soluble factors and metabolites in the lesion provide a unique microenvironment for fibrogenesis 15. Under this condition, stressed and injured tubular cells secrete pro-inflammatory factors, which in turn cause further tubular damage either directly or indirectly by recruiting inflammatory cells 16, 17. Damage-associated molecular pattern molecules (DAMPs) produced by injured cells are important links for the regulation of endogenous immune responses and repair processes 18. In the kidney, various DAMPs are activated upon acute injury and released by injured cells as danger signals, including high-mobility group box 1 (HMGB1) 19 and heat shock proteins (HSPs) 20.

HMGB1 is a ubiquitous DNA binding protein that may act as a hybrid sensor to drive nucleic acid-mediated immune responses 21. Under normal circumstances, HMGB1 is mainly present in the nucleus in a non-acetylated form. Upon stress, HMGB1is acetylated or phosphorylated, and then translocates to the cytoplasm for release into the extracellular space. Once released, HMGB1 turns into a DAMP molecule that activates innate immune receptors and drives inflammatory responses via the Nuclear factor-κB (NF-κB) signaling pathway 22. HMGB1 acetylation is thought to be conducted by a lysine acetyltransferase recruited by phosphorylated signal transducer and activator of transcription 1 (STAT1) dimers bound to the DNA 23.

In kidneys, HMGB1 has been implicated in acute kidney injury 24-27, sepsis on CKD 28, diabetic nephropathy 29, and free light chains-induced kidney injury 30, where it acts as a DAMP that amplifies inflammation and accelerates kidney damage. HMGB1 may also exacerbate tubulointerstitial fibrosis by skewing macrophage differentiation towards the M1 phenotype at early stages of obstructive kidney injury 31. Despite these studies, the involvement of HMGB1 in maladaptive repair following kidney injury is unclear. The present study aimed to investigate the role and regulation of HMGB1 during maladaptive repair following cisplatin nephrotoxicity. We demonstrate the aberrant activation of HMGB1 in post-RLDC kidneys, which further induces NF-κB signaling and consequent inflammation. Mechanistically, we show that HMGB1 activation is mediated by STAT1. Inhibition of STAT1 attenuates HMGB1 activation and ameliorates chronic kidney inflammation. These findings highlight the STAT1/HMGB1/NF-κB pathway as a new therapeutic target in cisplatin nephrotoxicity.

## Methods

### Animal models

All animal experiments were performed according to a protocol approved by the Institutional Committee for the Care and Use of Laboratory Animals of the Second Xiangya Hospital at Central South University. Male C57BL/6 mice (8 weeks of age) were purchased from SJA Laboratory Animal Corporation (Changsha, Hunan, China) and maintained in the standard pathogen-free animal facility of the Second Xiangya Hospital under a 12:12 h light-dark cycle with free access to food and water. Mice received 8 mg/kg cisplatin (Hansoh Pharma, Jiangsu, China) or saline as control via intraperitoneal injection once a week for 4 weeks as previously 8. For intervention, at 7 days after the last dose of cisplatin, animals were randomly divided for intraperitoneal injection of anti-HMGB1 neutralizing antibody (Ab) or non-immune Ig (Shino-Test Co, Sagamihara-shi, Kanagawa, Japan). This anti-HMGB1 Ab was characterized *in vitro* and used at 200 μg per mouse for *in vivo* studies as before 19. Animals were sacrificed at 1 and 4 weeks after the last cisplatin injection to collect samples.

### Cells and cisplatin treatment

The Boston University mouse proximal tubular cell line (BUMPT) was originally obtained from Dr. Lieberthal (Boston University) and cultured as previously 32. BUMPT cells were subjected to four cycles of treatment with 0, 0.5, 1, or 2 μM cisplatin 8. Small interference RNA (siRNA) specific against the mouse HMGB1 gene (si-*Hmgb1-1* GGAGAGATGTGGAACAACA, si-*Hmgb1-2* GGATATTGCTGCCTACAGA), STAT1 gene (si-*Stat1-1* CTGTGATGTTAGATAAACA, si-*Stat1-2* GCAGCACAACATACGGAAA) and negative control (NC) were synthesized by RiboBio (Guangzhou, China). Cells were transfected with 50 nM siRNA or NC siRNA for 24 h using riboFECT_TM_ CP Transfection Kit (RiboBio, China). Fludarabine is an inhibitor of STAT1 that specifically reduces STAT1 without affecting other STAT family members 33. In this study, fludarabine (Selleck, Houston, TX) were used at 50, 75, or 100 μM for cell treatment.

### Transcutaneous Measurement of glomerular filtration rate (GFR)

GFR was measured in mice by transcutaneously monitoring the clearance of fluorescein isothiocyanate (FITC) - labeled sinistrin. Briefly, mice were anesthetized with isoflurane. Then a double-sided adhesive patch was used to adhere the transdermal GFR monitor (MediBeacon, Mannheim, Germany). FITC-sinistrin (7 mg /100g b.w.) was injected via a tail vein. After 1 h, the devices were removed, and data were analyzed using elimination kinetics curve of FITC-sinistrin as previously described 34.

### Histological and immunohistological analysis

Hematoxylin and eosin (HE) staining were conducted using a standard protocol as previously described 8. Tubular damage was evaluated on a scale of 0-4, which ranged from absent (0) to mild (1), moderate (2), severe (3), and very severe injury (4). Masson trichrome staining was performed using the reagents from Servicebio (Wuhan, China). For quantification, 10-20 positive collagen-stained fields (100 magnification) were randomly selected from each section and analyzed by Image-Pro Plus 6.0. The blue-stained area ratio to the area of the entire field (glomeruli, tubule lumina, and blood vessels, if any, excluded) was assessed and expressed as a percentage of the fibrotic area. And for immunohistochemical staining, it was performed as previously 8. For quantification, randomly selected 10-20 fields from each tissue section were evaluated to determine the percentage of positive staining cells per square millimeter.

### Immunofluorescence

BUMPT cells seeded on glass coverslips were fixed in cold methanol: acetone (1:1) at -20 °C for 10 min. After further washing with phosphate buffered saline, the fixed cells were blocked in 10% normal goat serum for 45 min. The cells were then incubated with the primary antibody overnight, and with goat anti-rabbit IgG at 37 °C for 1 h. The cells were counter-stained with Hoechst (Beyotime, Shanghai, China) to show the nucleus. Finally, the slides were examined on the Zeiss LSM780 confocal microscope (Oberkochen, Germany). For fluorescence intensity statistics, Image Pro Plus was used to count the nuclear fluorescence intensity (IOD/AREA) of the selected area of a single cell and the fluorescence intensity of the cytoplasmic selected area, and finally calculate the ratio of the two.

### Extraction of nuclear and cytoplasmic proteins

Cytoplasmic and nuclear proteins were extracted with the nuclear and cytoplasmic protein extraction kit (Beyotime, Shanghai, China). After treatment, cells were washed with PBS and scraped to collect the cell pellet by centrifugation at 2000 g for 5 min. The cell pellet (20 μL) was mixed with PMSF-containing cytoplasmic protein extraction reagent A (200 μL), vortexed vigorously for 5 s, and incubated in ice bath for 10-15 min. Cytoplasmic protein extraction reagent B (10 μL) was then added. After 5 s vigorous vortexing and ice bathing for 1 min, the mixture was centrifuged at 12000 g for 5 min at 4 ºC to collect the supernatant as the cytoplasmic fraction. Then, the pellet was re-suspended in the nucleoprotein extraction reagent (50 μL) by vigorous vortexing (15-30 s every 1-2 min for a total of 30 min), and then centrifuged at 12000 g for 10 min at 4 ºC to collect the supernatant as the nuclear proteins.

### Immunoblot analysis

2% SDS buffer with 1% protease inhibitor cocktail (P8340, Sigma-Aldrich) was used to lyse renal cortex and outer medullary tissue. The protein concentration was determined using the Pierce BCA Protein Assay Kit from Thermo Scientific. The primary antibodies used for immunoblotting included: anti-Fibronectin (ab2413), anti-α-Smooth Muscle Actin (ab5694), anti-IL-6 (ab229381) and anti-TNF-α (ab183218) from Abcam; anti-STAT1(D1K9Y) (14994), anti-phospho-STAT1(Y701) (9167), anti-HMGB1(D3E5) (6893), anti-NF-κB p65 (8242), anti-phospho-NF-κB p65 (3033), anti-GAPDH (5174), anti-Vimentin (5741), anti- Histone H3 (1B1B2) (14269) and anti-IL-1β (12242) from Cell Signaling Technology; anti-Collagen type I (AF7001) from Affinity. anti- Lamin B (12987-1-AP) from Proteintech. All secondary antibodies for immunoblot analysis were from Thermo Fisher Scientific (MA, USA). The samples were resolved on SDS-polyacrylamide gel, and then transferred for immunoblotting. The PVDF membrane is incubated with 5% BSA for 1 h. The membranes were then incubated overnight with primary antibodies according to the ratios in the instructions. After rewarming, the secondary antibody was incubated for 1 h. Finally, the chemiluminescence imaging system was used for luminescence imaging. For densitometry, the protein bands were analyzed with the NIH ImageJ software.

### Co‑immunoprecipitation of HMGB1 and TLR4

After RLDC treatment, BUMPT cells were lysed in ice-cold immunoprecipitation lysis buffer (150 mM NaCl, 5% glycerol, 50 mM Tris HCl pH 8.0, 1 mM MgCl_2_, 1.0% NP40) with 1% protease inhibitor cocktail on ice for 30 min. After centrifugation at 12,000 rpm for 10 min at 4 °C, the supernatants were incubated with anti-HMGB1 (6893, Cell Signaling Technology) (1:150 dilution), anti-TLR4 antibody (66350-1-Ig, Proteintech) (1:100 dilution), or IgG (1:150 dilution) as control overnight at 4 °C, and the antigen-antibody complexes were then precipitated by the Protein A/G PLUS-Agarose (sc-2003, Santa-Cruz). The immunoprecipitation complexes were washed, eluted, and then analyzed by immunoblot analysis.

### Quantitative real-time PCR

Total RNA from kidney tissues and cells was extracted with TRIzol reagents (CWBIO, Jiangsu, China) according to the manufacturer's protocol. cDNA was synthesized using the PrimeScript RT reagent Kit (TaKaRa, Japan). Quantitative real-time PCR was performed using the TB Green Premix Ex Taq II reagent (TaKaRa, Japan) on a LightCycler96 Real-Time PCR System. Relative expression was normalized to the expression levels of GAPDH. The primer sequences used for PCR are shown in Table S1.

### ChIP assay

ChIP assay was performed with the ChIP-IT® High Sensitivity kit (Active Motif, USA) according to the manufacturer's instruction. Briefly, cells were cross-linked with 1% formaldehyde followed by incubation in glycine stop-fix solution. Then, the cells were lysed with the lysis buffer containing protease inhibitor cocktail and deacetylase inhibitors. Purified chromatin was sonicated and incubated with an indicated immunoprecipitation antibody anti-STAT1 antibody (Cell Signaling Technology, 14994). The resultant immune-precipitate was subjected to qPCR amplification of putative Stat1 binding sequences using specifically designed primers. The value of qPCR was normalized with input DNA for comparison. The following primers were used for STAT1 binding site 1: forward CATGCCAATGATATTCACGA and reverse TATGTAATGGATTTGCTCGAA, STAT1 binding site 2: forward GTCACAGAAAACAGCGTCT and reverse CTGTTTTGAACTTCCCGGACA.

### GEO database acquisition and analysis

We retrieved transcriptional expression profiles of renal biopsy specimens from CKD patients (non-CKD samples: 8; CKD samples: 54) deposited in Gene Expression Omnibus (GEO) under the accession number GSE66494 35. The studied STAT1 and HMGB1-related gene sets were downloaded from the Molecular Signatures Database of (Gene Set Enrichment Analysis) GSEA (https://www.gsea-msigdb.org/gsea). We used R statistical software (version 4.0.3) and “limma” package to perform significance analysis of differentially expressed gene (DEGs) between non-CKD and CKD renal tissues in the GSE66494 dataset. The adjusted p-value < 0.05 and transcripts with a fold expression greater than 2 were used as the cutoff for difference analysis. Gene Ontology (GO) enrichment analysis was performed using the R packages “clusterProfiler” to explore the functions and pathways of differentially expressed genes. A value of P < 0.05 was considered to be statistically significant. After log2 conversion, the DEGs between the normal group and the CKD group were visualized using heat maps drawn with the R package "pheatmap".

### Clinical pathology, evaluation of HMGB1/NF-κB in human CKD renal biopsy samples

Paraformaldehyde-fixed, paraffin-embedded renal biopsy samples were sectioned at 3 µm. The slides were reviewed by 2 independent pathologists and a nephrologist in a blinded manner. Interstitial fibrosis and tubular atrophy (IFTA) score was determined by tubular injury (brush border loss, necrosis, atrophy, etc), interstitial inflammation and excessive collagen deposition according to the Banff working classification 36. Next, immunofluorescence staining was performed on paraffin sections and counted. Briefly, 10 randomly selected areas (400×) from each section were counted for IOD values using Image pro plus and the IOD value is divided by the area to obtain Average Density. For p65 staining, 10 areas (400×) were randomly selected from each section and the number of p65+ nucleus was counted, and the mean value was subsequently obtained.

### Statistics

Statistical analysis was performed using the GraphPad Prism software. For multiple group comparison, ANOVA followed by Tukey's posttests was used. Statistical differences between two groups were determined by two-tailed unpaired or paired Student's t-test. For GEO database analysis, strawberry Perl for windows (Version5.18.2) was used for data processing, and R (4.1.1) was performed for data analysis. The “pheatmap” package was used to conduct hierarchical clustering analysis. P < 0.05 was considered statistically significant.

## Results

### HMGB1 is upregulated in mouse kidneys after RLDC treatment

**Figure 1:**
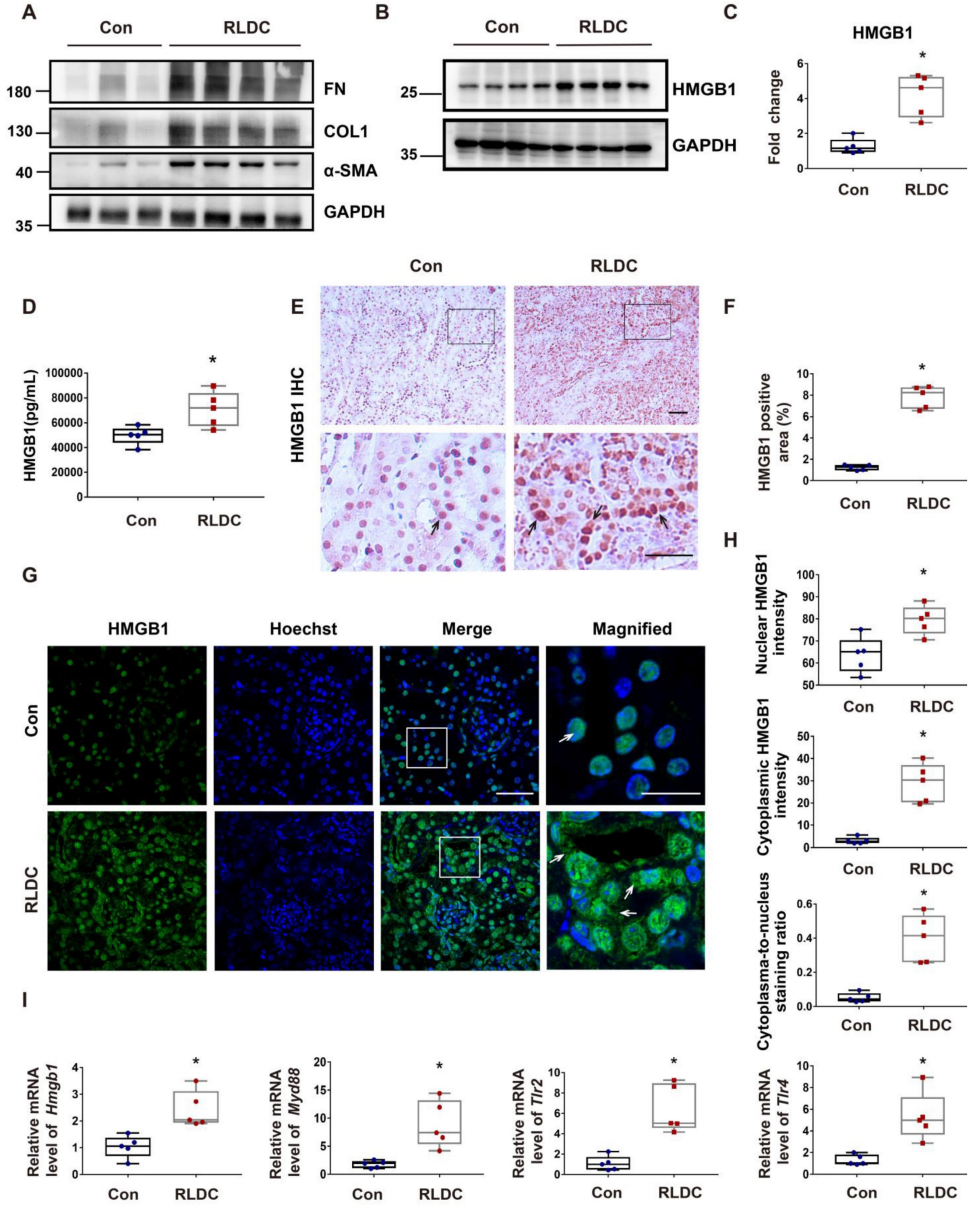
** HMGB1 is upregulated after RLDC treatment.** C57BL/6 mice were injected weekly with 8 mg/kg cisplatin for four weeks and sacrificed for analysis one month later (RLDC group) or left untreated as controls (Con) to collect kidneys for analysis. (A) Representative immunoblots of fibronectin (FN), collagen-1 (COL-1), smooth muscle alpha-actin (α-SMA), and GAPDH (loading control) in kidney tissues. (B) Representative immunoblots of HMGB1 and GAPDH. (C) Densitometric analysis of HMGB1 immunoblots. (D) Quantitative analysis of serum HMGB1 by ELISA. (E) Representative images of immunohistochemical staining of HMGB1 in kidney tissues. The arrows point to nuclear staining of HMGB1 in control or nuclear and cytoplasmic staining in RLDC kidneys. Bar scale = 50 μm. (F) Quantification of HMGB1 positive areas on kidney sections. (G) Representative images of HMGB1 immunofluorescence (green) and Hoechst nuclear staining (blue) in kidney tissues. Scale bar = 50 μm; scale bar in the magnified images = 25 μm. (H) Quantitative analysis of HMGB1 staining in tubular cells. (I) qRT-PCR analysis of *Hmgb1*,* Myd88*,* Tlr2*,* and Tlr4* mRNAs in control and RLDC kidneys. The expression of the target genes was normalized to GAPDH mRNA and expressed as fold change compared to control kidneys (Con). Quantitative data are expressed as mean ± SEM. N=5. *P < 0.05 vs. the control group (Con).

To investigate the role of HMGB1 in RLDC-induced chronic inflammation and renal fibrosis, we analyzed HMGB1 expression. One month after RLDC treatment, several fibrosis markers, including fibronectin (FN), collagen 1 (COL1), and smooth muscle alpha-actin (α-SMA), were upregulated in kidney tissues (Figure 1A). This fibrotic phenotype correlated with an increase of about 4-fold in renal HMGB1 protein (Figure 1B-C). Since HMGB1 can be secreted and act as pro-inflammatory cytokine, we also assessed serum HMGB1 level in RLDC-treated and control mice. Serum HMGB1 in RLDC-treated mice was twice as high as in control mice (Figure 1D). In immunohistochemistry, HMGB1 was mainly detected in the nucleus of renal tubular cells in the control mice. After RLDC treatment, remarkable increases in HMGB1 were visible in both the cytoplasm and nucleus of renal tubule cells (Figure 1E-F). These results were also confirmed by immunofluorescence (Figure 1G). To further validate these observations, we quantified the HMGB1 immunofluorescence signals in the nucleus and in the cytoplasm of tubular cells, and calculated the cytoplasma-to-nucleus staining ratio (Figure 1H). This analysis demonstrated that RLDC markedly affected the level and distribution of HMGB1 in renal tubular cells. To determine whether the pathways acting downstream of HMGB1 were also activated, we assessed the transcriptional regulation of *Hmgb1* and its downstream signaling genes including (myeloid differentiation factor 88) *Myd88,* (Toll-like receptor) *Tlr2,* and* Tlr4*. As shown in Figure 1I, mRNA expression of HMGB1 and its downstream signaling genes was up-regulated in the kidney after RLDC treatment, suggesting a role of the HMGB1 pathway in RLDC-induced kidney problems.

### HMGB1 is induced by RLDC in cultured renal tubular cells

**Figure 2:**
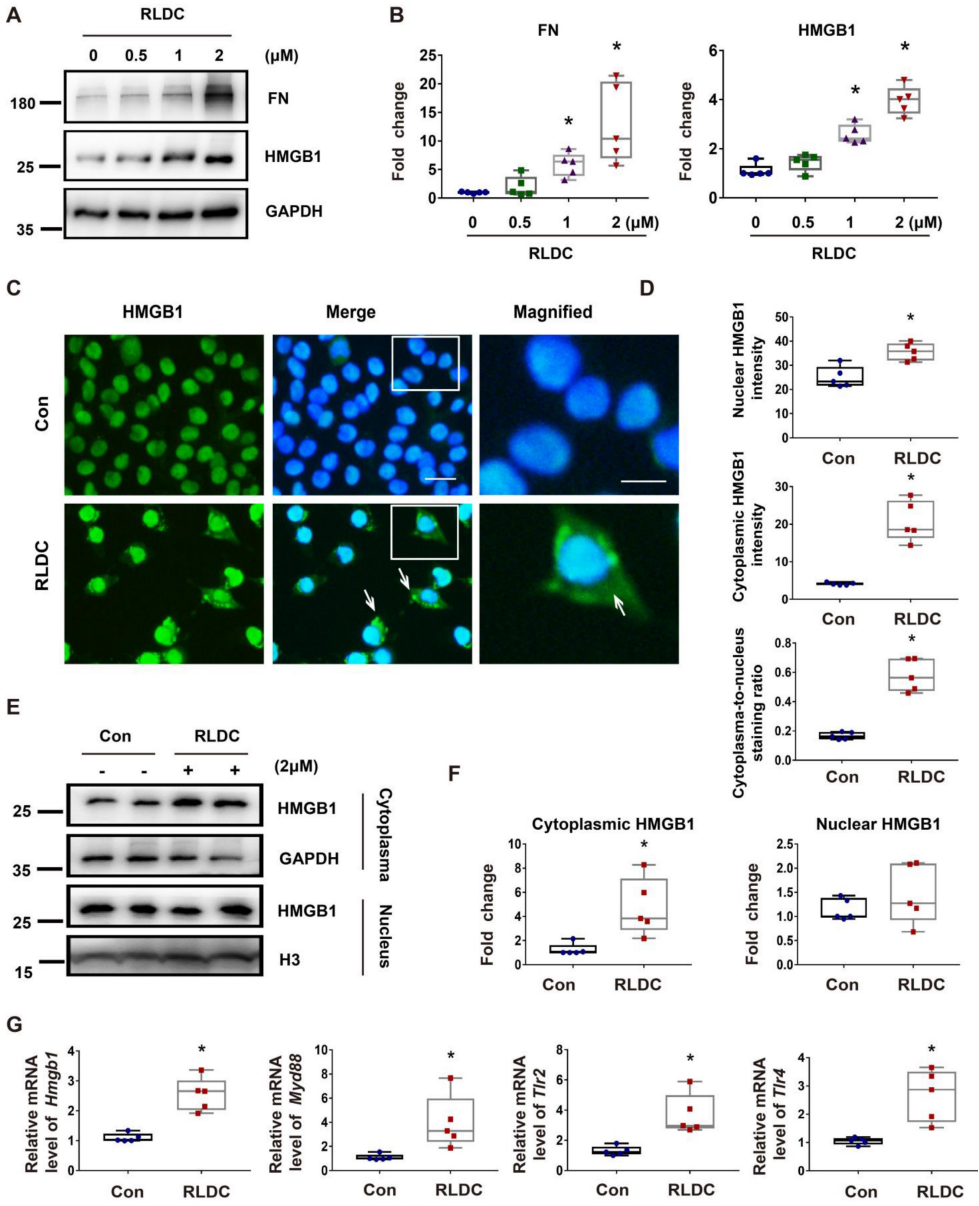
** HMGB1 is induced by RLDC treatment of cultured renal tubular cells.** BUMPT cells were incubated with cisplatin for 7 h every day for four days or left untreated (Con or 0 μM), and collected at day 5. (A) Representative immunoblots of fibronectin (FN), HMGB1, and GAPDH (loading control). (B) Densitometric analysis of the immunoblots in A. (C) Immunofluorescence analysis of HMGB1 (green) translocation. The nuclei were stained with Hoechst (blue). The arrows indicate cytoplasmic HMGB1 in RLDC-treated cells. Scale bar = 20 μm; scale bar in magnified images = 10 μm. (D) Quantitative analysis of HMGB1 staining in BUMPT cells. (E) Immunoblots of HMGB1 in the nuclear and cytosolic fractions of untreated (Con) and RLDC-treated BUMPT cells. Histone 3 protein (H3) and GAPDH were used as internal controls for nuclear fractions and whole cell lysate, respectively. (F) Densitometric analysis of the immunoblots in E. (G) qRT-PCR analysis of *Hmgb1*, *Myd88*, *Tlr2*, and *Tlr4* mRNAs. The expression of the target genes was normalized to GAPDH mRNA and expressed as fold change compared to control cells (Con). Quantitative data are expressed as mean ± SEM. N = 5. * P <0.05 vs. the control group (Con).

Next, we investigated whether and how HMGB1 is involved in RLDC-induced renal fibrosis by using a well-established cell model 8. RLDC increased HMGB1 expression in BUMPT mouse kidney proximal tubular cells, which was accompanied by an accumulation of the fibrosis marker protein FN in a dose-dependent manner (Figure 2A-B). Importantly, in addition to increased expression, HMGB1 showed a partial localization in the nucleus upon RLDC treatment (Figure 2C). HMGB1 signal intensity in the cytoplasm of RLDC-treated cells increased about 5-fold compared to that in untreated cells (Figure 2D). The staining result was further confirmed by immunoblot analysis of the cytosolic and nuclear fractions of the cells. As show in Figure 2E and 2F, a significant portion of HMGB1 appeared in the cytosolic fraction in RLDC-treated cells. Consistent with the *in vivo* findings shown in Figure 1, RLDC induced mRNA expression of HMGB1 and its receptors in BUMPT cells, indicative of transcriptional activation (Figure 2G).

### Blockade of HMGB1 protects against renal and tubular cell injury and fibrotic phenotype after RLDC

**Figure 3:**
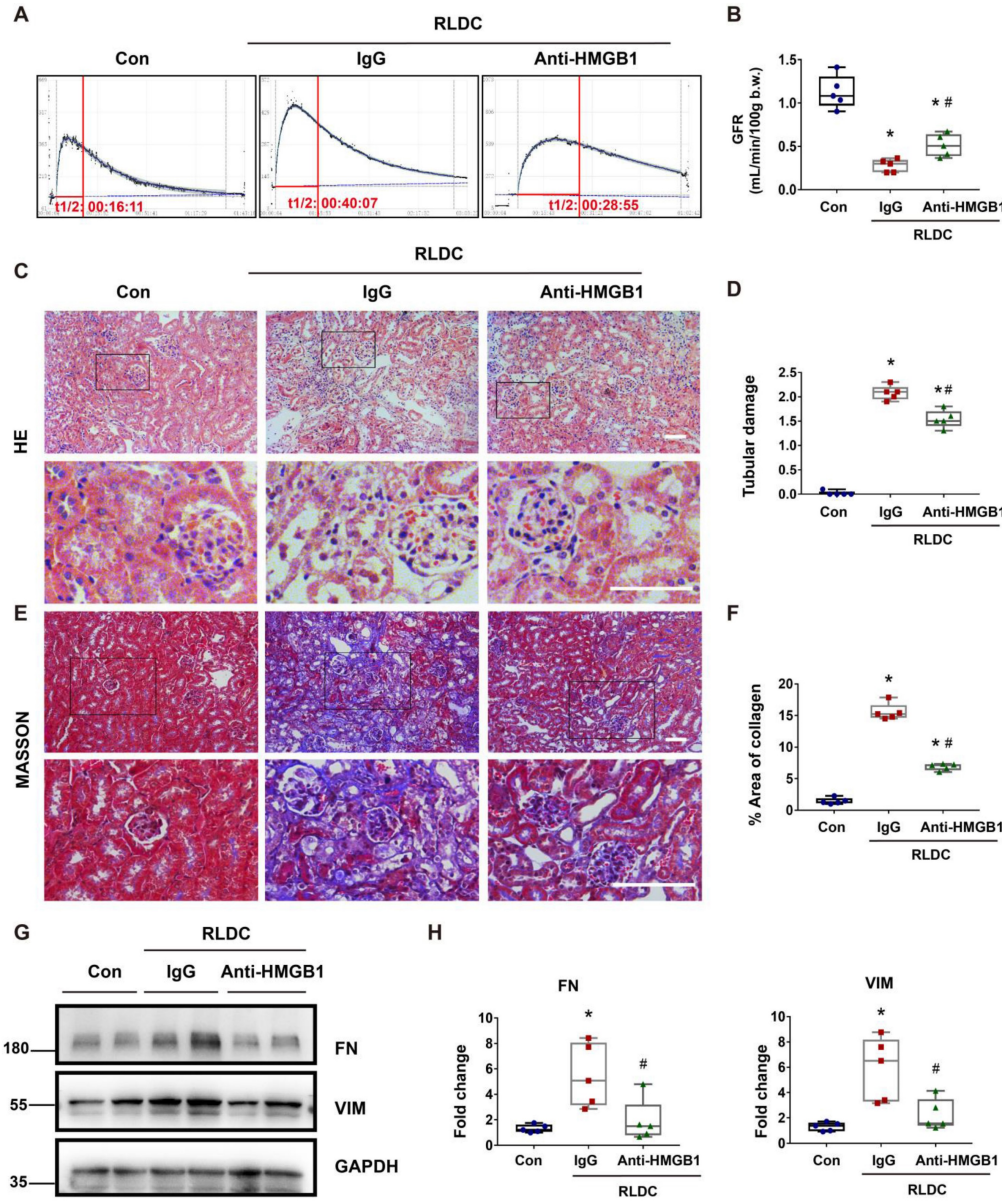
** Anti-HMGB1 neutralizing antibody protects against post-RLDC kidney injury and fibrosis.** C57BL/6 mice received a weekly injection with 8 mg/kg cisplatin for four weeks. After the last cisplatin injection, anti-HMGB1 antibody or control IgG were injected daily for one week for sample collection and analysis. (A) Representative tracing of FITC-sinistrin clearance to indicate glomerular filtration rate (GFR). (B) Summary of GFR measured by FITC-sinistrin clearance. (C) Representative renal histology of hematoxylin and eosin (HE)-staining showing the beneficial effect of anti-HMGB1 antibody in RLDC-treated mice. Scale bar = 50 μm. (D) Kidney tubule damage score. (E) Representative images of Masson's trichrome staining of kidney tissues. Scale bar = 50 μm. (F) Quantification of the collagen positive area according to MASSON staining. (G) Representative immunoblots of FN, VIM and GAPDH in kidney tissues. (H) Densitometric analysis of FN and VIM protein levels in immunoblots. Quantitative data are expressed as mean ± SEM. N = 5. * P <0.05 vs. the untreated control group (Con); #* P* < 0.05 vs. RLDC/IgG group.

To determine the role of HMGB1 in post-RLDC renal problems, we tested the effects of HMGB1 neutralizing antibody (Anti-HMGB1) or non-immune IgG (IgG) that were given to mice for 1 week after RLDC treatment. Functionally, RLDC induced a marked decrease in glomerular filtration rate (GFR). However, this decrease was partially but significantly prevented by anti-HMGB1, in comparison with non-immune IgG (Figure 3A-B). Histological evaluation further confirmed that anti-HMGB1 protected against renal tubule damage, which was manifested by tubular necrosis, brush border loss, cast formation, and tubular dilatation in renalcortex and outer medulla (Figure 3C-D). In addition, the neutralizing antibody alleviated renal interstitial fibrosis in post-RLDC kidneys as shown by Masson trichrome staining and immunoblot analysis of fibrosis proteins (Figure 3E-H). We also evaluated the effect of Glycyrrhizin (GZ), a specific pharmacological inhibitor of HMGB1. The results show that GZ provided protection against renal fibrosis and also reduced the extent of tubular damage. In addition, GZ suppressed p65 phosphorylation and nuclear accumulation (Figure S1). Together, these data support an important role of HMGB1 in the development of kidney problems after cisplatin nephrotoxicity.

To further explore the role of HMGB1 in renal fibrosis, we examined the effects of *Hmgb1* knockdown in BUMPT cells during RLDC treatment. HMGB1 knockdown cells were generated by using two different *Hmgb1* siRNAs named siH1-1 and siH1-2, while control cells were transfected with a negative control siRNA (siNC) (Figure S2 A-B). RLDC treatment increased the expression of FN, which was significantly decreased in *Hmgb1*-knockdown cells (Figure S2 C-D). Immunostaining further confirmed that RLDC-induced COL-1 expression was decreased almost by half by HMGB1 knockdown (Figure S2 E-F). Together, these data indicate that HMGB1 contributes to the development of the profibrotic phenotype in renal tubular cells during RLDC treatment.

### Anti-HMGB1 neutralizing antibody suppresses NF-κB activation in post-RLDC kidneys

**Figure 4:**
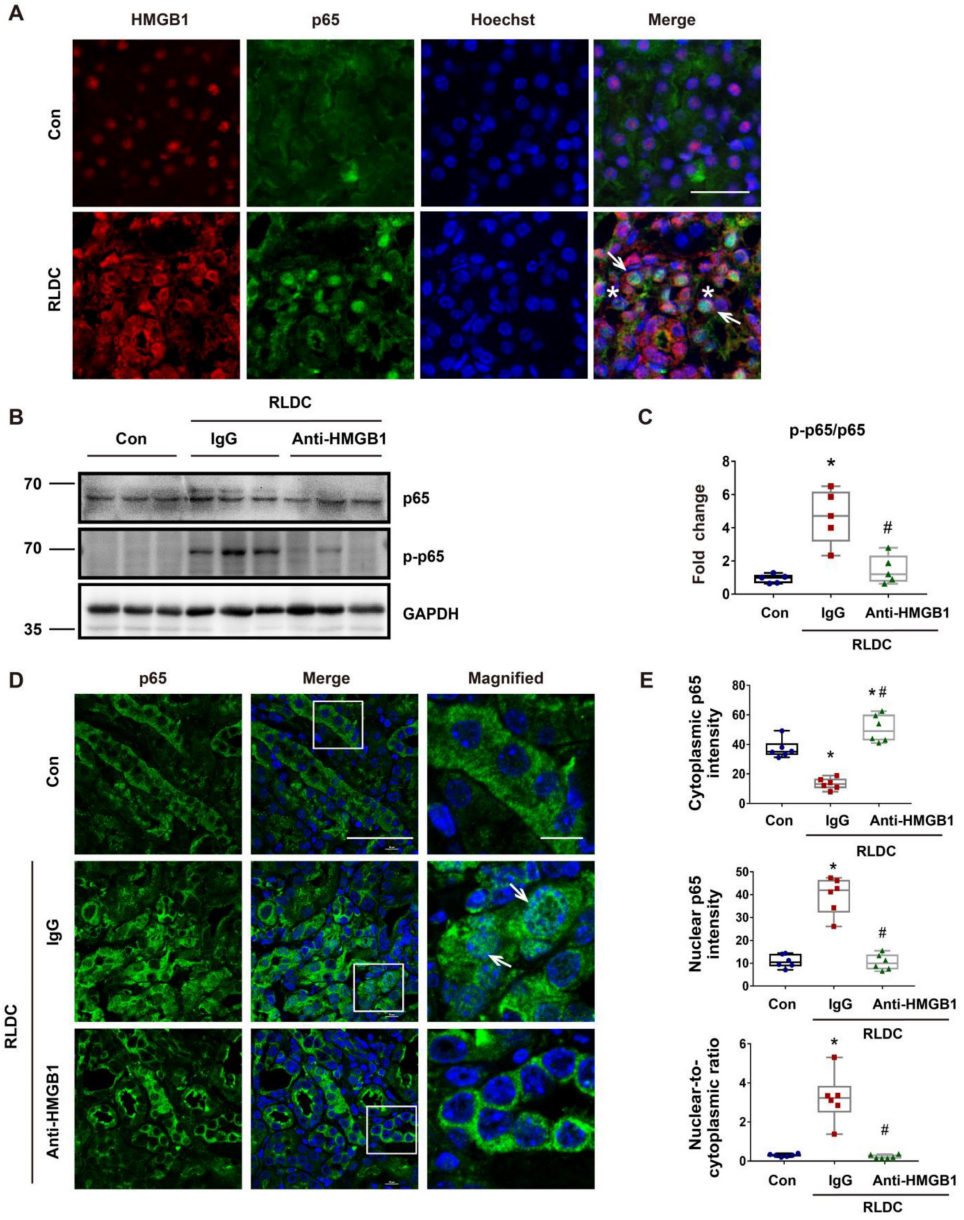
** Anti-HMGB1 neutralizing antibody suppresses NF-κB activation in post-RLDC kidneys.** C57BL/6 mice received a weekly injection with 8 mg/kg cisplatin for four weeks. After the last injection, anti-HMGB1 antibody or control IgG were injected daily for one week for sample collection and analysis. (A) Immunofluorescence co-staining of HMGB1 (red) and p65 (green) with Hoechst staining of the nucleus (blue) in kidney tissues of control (Con) or RLDC--treated mice (RLDC). Arrows point to representative tubule cells with cytoplasmic HMGB1 and nuclear p65. Asterisks indicate the lumen of damaged tubules. Scale bar = 20 μm. (B) Representative immunoblots of p65, phosphorylated p65 (p-p65), and GAPDH (loading control). (C) Densitometry of p-p65/p65 ratio in immunoblots. (D) Analysis of p65 nuclear translocation by immunofluorescence and laser-scanning confocal microscopy. Arrows point to the cells with nuclear p65. Scale bar = 50 μm; scale bar on enlarged pictures = 10 μm. (E) Quantitative analysis of cytoplasmic p65, nuclear p65 and their ratio. N = 5. * P <0.05 vs. the control group (Con), #* P* < 0.05 vs. RLDC/IgG group.

We next investigated the mechanism whereby HMGB1 participates in RLDC-induced maladaptive kidney repair. NF-κB signaling is a major pathway for inflammation by driving the expression of a variety of pro-inflammatory cytokines, including those in kidneys 37. Notably, NF-κB has been implicated in HMGB1-mediated danger signal activation 38. We therefore hypothesized that HMGB1 could induce pro-inflammatory factors in renal tubular cells through NF-κB. To test this, we first analyzed the expression and localization of HMGB1 and the p65 subunit of NF-κB in kidneys by immunofluorescence. In control kidneys, both HMGB1 and p65 had weak staining, and while HMGB1 was mainly localized in the nucleus, p65 was in the cytoplasm (Figure 4A).

RLDC treatment induced both HMGB1 and p65, and notably, HMGB1 translocated into the cytoplasm in some cells, while p65 accumulated into the nucleus. During NF-κB activation, p65 is phosphorylated and translocated into the nucleus. To investigate the possible regulation of NF-κB signaling by HMGB1, we analyzed the effect of HMGB1 neutralizing on p65 phosphorylation (p-p65) during RLDC treatment. As shown in Figure 4B and C, RLDC induced p-p65 in kidneys, which was suppressed by HMGB1 neutralizing antibodies. Immunofluorescence also demonstrated p65 accumulation in the nucleus of renal tubular cells, which was attenuated by HMGB1 neutralizing antibodies (Figure 4D). Consistently, HMGB1 neutralizing antibodies provoked a re-localization of nuclear p65 to the cytoplasm, as evidenced by the decrease in the nuclear-to-cytoplasmic ratio of p65 (Figure 4E). Next, we assessed the effect of HMGB1 neutralization on the transcription of downstream cytokines and chemokines of the NF-κB pathway. Mice receiving HMGB1 neutralizing antibodies after RLDC had significantly less *Il-1β*, *Il-6*, and *Tnf-α* expression than those receiving non-immune IgG (Figure S3). Collectively, these data indicate that HMGB1 plays a pivotal role in the activation of NF-κB signaling for inflammation during the development of chronic kidney problems after cisplatin nephrotoxicity.

### *Hmgb1* knockdown decreases NF-κB activation in RLDC-treated renal tubular cells

**Figure 5:**
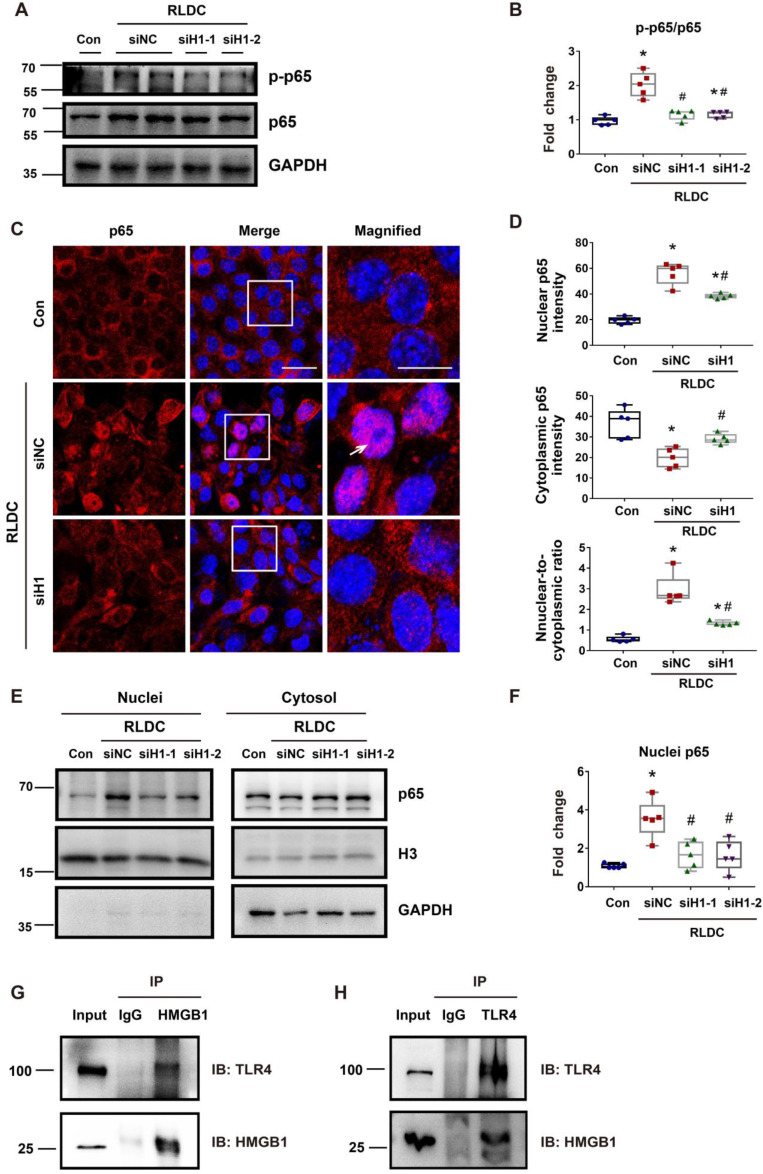
***Hmgb1* knockdown decreases NF-κB activation in RLDC-treated renal tubular cells.** BUMPT cells were transfected with 50 nM of HMGB1 siRNA1 (siH1-1), HMGB1 siRNA2 (siH1-2), or a negative control siRNA (siNC), and then subjected to 4 days of RLDC treatment or control incubation (Con). (A) Representative immunoblots of p-p65, p65, and GAPDH (loading control). (B) Densitometry of p-p65/p65 ratio in immunoblots. (C) Representative immunofluorescence of p65 (red) with Hoechst staining of nuclei (blue) showing p65 nuclear translocation in RLDC treatment that was suppressed by si*Hmgb1*. Scale bar = 20 μm; scale bar on enlarged images = 10 μm. Arrows indicate cells with nuclear p65. (D) Quantitative analysis of cytoplasmic p65, nuclear p65, and their ratio. (E) Representative immunoblots of nuclear and cytoplasmic p65. (F) Densitometry of nuclear p65. (G) and (H) Co-IP analysis of HMGB1 and TLR4 interaction. After RLDC treatment, BUMPT cells were lysed in immunoprecipitation lysis buffer with protease inhibitor. After immunoprecipitation, the complexes were washed, eluted, and analyzed by immunoblot analysis. Quantitative data are expressed as mean ± SEM. N = 5. * P <0.05 vs. control group without RLDC treatment (Con); #* P* < 0.05 vs. RLDC/siNC cells.

To further confirm the regulation of NF-κB by HMBG1 in renal tubular cells, we knocked down *Hmgb1* with two specific siRNAs (siH1-1, siH1-2). Both *Hmgb1* siRNAs attenuated p65 phosphorylation during RLDC treatment (Figure 5A-B). They also reduced p65 accumulation in the nucleus (Figure 5C). Quantitatively, RLDC induced a 2 to 3-fold increase in nuclear p65, which was diminished in *Hmgb1* siRNA cells (Figure 5D). The staining result was confirmed by immunoblot analysis of p65 in the nuclear and cytosolic fractions (Figure 5E-F). Moreover, *Hmgb1* knockdown suppressed the expression of pro-inflammatory cytokines such as *Il-1β*, *Il-6*, and *Tnf-α* in RLDC-treated cells (Figure S4). To understand how HMGB1 activates NF-κB signaling, we found that tubular cells RLDC induced the expression of TLR4 and MyD88 (Figure 2G). TLR4 is the main receptor of HMGB1 that relays signals for NF-κB activation 39. In co-immunoprecipitation (Co-IP) assay, we detected the binding of HMGB1 to TLR4 in RLDC-induced cells (Figure 5G-H). Together, these results indicate that HMGB1 may contribute to NF-κB activation during RLDC treatment in renal tubular cells through TLR4.

### STAT1 mediates RLDC-induced cytoplasmic accumulation of HMGB1 in renal tubular cells

**Figure 6:**
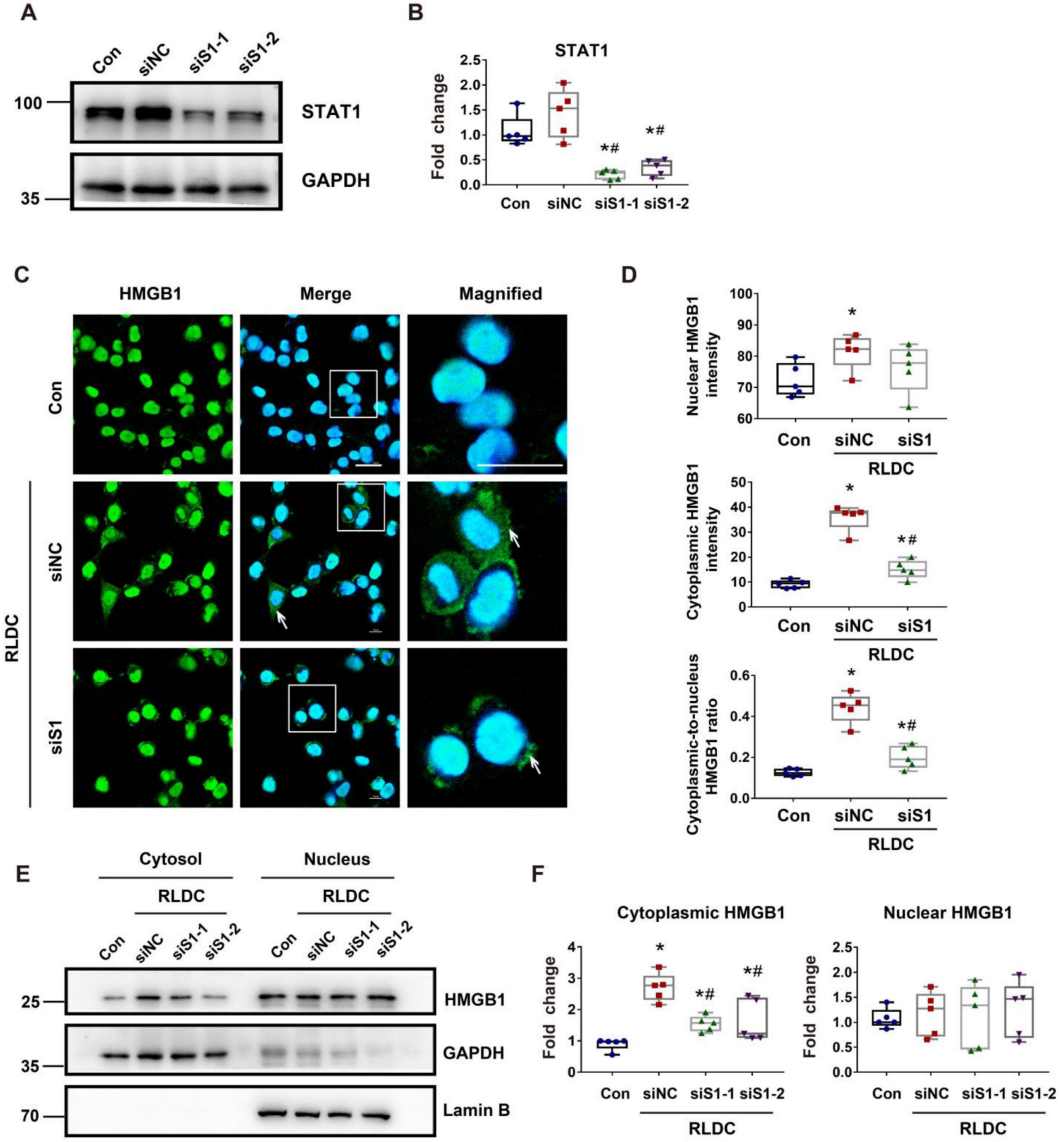
** STAT1 regulates RLDC-induced cytoplasmic accumulation of HMGB1 in renal tubular cells.** BUMPT cells were transfected with 50 nM STAT1 siRNA1 (siS1-1), siRNA2 (siS1-2), or negative control siRNA (siNC), and then subjected to 4 days of RLDC treatment or left untreated (Con). (A) Representative immunoblots of STAT1 and GAPDH (loading control). (B) Densitometry of STAT1. (C) Analysis of HMGB1 translocation by immunofluorescence and laser-scanning confocal microscopy; HMGB1 (green), nuclear staining with Hoechst (blue). Scale bar = 20 μm. (D) Quantitative analysis of nuclear and cytoplasmic HMGB1 signals and the nuclear-to-cytoplasmic ratio. (E) Representative immunoblots of nuclear and cytoplasmic HMGB1. Lamin B and GAPDH were used as internal controls for the nuclear and cytosolic factions, respectively. (F) Quantitative analysis of immunoblots of nuclear and cytoplasmic HMGB1. For calculation, the protein level in the control culture was arbitrarily set to 1, and the signals of other conditions were normalized with control to calculate fold changes. Data are expressed as mean ± SEM. N = 5. * P<0.05 vs. the control group (Con), #* P* < 0.05 vs. RLDC/siNC group.

**Figure 7:**
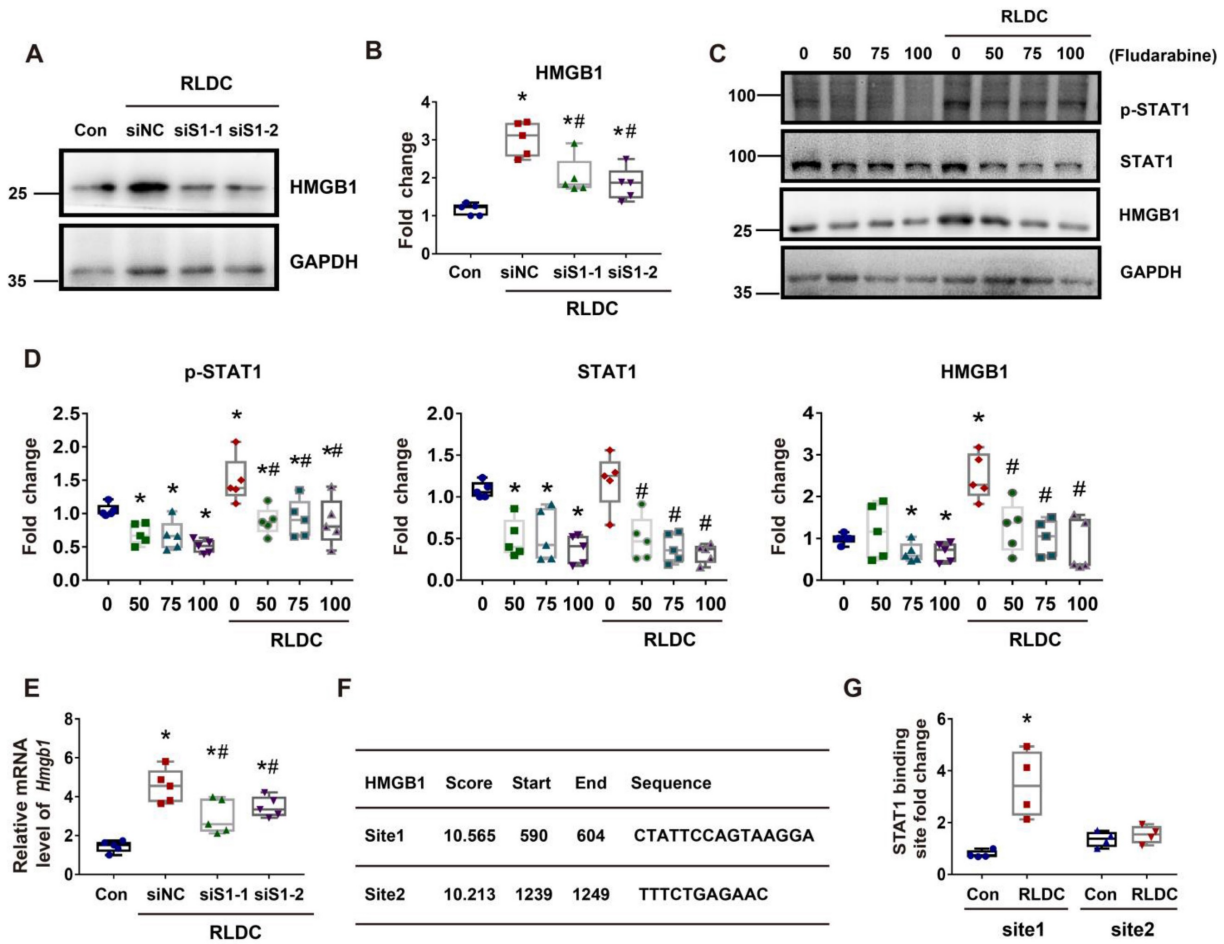
** STAT1 transcriptionally mediates HMGB1 upregulation during RLDC treatment.** BUMPT cells were transfected with 50 nM STAT1 siRNA1 (siS1-1), siRNA2 (siS1-2), or negative control siRNA (siNC), and then subjected to 4 days of RLDC treatment or left untreated (Con). For drug intervention, after RLDC treatment, the cells were incubated with 50, 75, or 100 μM fludarabine for 17 h. (A) Representative immunoblots of HMGB1 and GAPDH (loading control) to verify *Stat1*-knockdown. (B) Densitometry of HMGB1 immunoblots. (C) Representative immunoblots of p-STAT1, STAT1, HMGB1 and GAPDH showing the effects of fludarabine. (D) Densitometry of p-STAT1, STAT1, and HMGB1 in blots. (E) qRT-PCR analysis of* Hmgb1* mRNA. The *Hmgb1* mRNA values were normalized to GAPDH mRNA and expressed as fold change compared to the control. (F) Predicted STAT1 binding site in HMGB1 promoter region using JASPAR database (http://jaspar.genereg.net/). (G) ChIP assay of STAT1 binding to HMGB1 promoter sequences. Quantitative data are expressed as mean ± SEM. N ≥ 4. * P <0.05 vs. the control group (Con), #* P* < 0.05 vs. RLDC/siNC cells or RLDC with 0 μM fludarabine cells.

Mechanistically, HMGB1 had two remarkable changes in RLDC-treated renal tubular cells, i.e. cytoplasmic accumulation and increased expression. STAT1 was shown to be a key mediator of the cytoplasmic accumulation of HMGB1 in stimulated macrophages 23. To determine the role of STAT1 in our model, we knocked down STAT1 with two specific *Stat1* siRNAs (siS1-1 and siS1-2) in BUMPT cells (Figure 6A-B). As shown in Figure 7C, RLDC-induced cytoplasmic accumulation of HMGB1 was largely attenuated in si*Stat1* cells, in comparison with the cells transfected with negative control siRNA (siNC) (Figure 6C). The quantification of cytoplasmic and nuclear HMGB1 signals further indicated that *Stat1* knockdown significantly blocked the release of nuclear HMGB1 into the cytoplasm, as shown by the reduction of the plasma-to-nucleus HMGB1 ratio (Figure 6D). We further analyzed nuclear and cytoplasmic HMGB1 in cellular fractionations by immunoblotting. As shown in Figure 6E and 6F, RLDC induced an around 3-fold increase of cytoplasmic HMGB1 in siNC cells, but only 1.5 fold in si*Stat1* cells. These results support a critical role of STAT1 in RLDC-induced HMGB1 redistribution in renal tubular cells.

### STAT1 mediates HMGB1 upregulation in RLDC-treat renal tubular cells transcriptionally

In addition to cytoplasmic accumulation, RLDC induced HMGB1 expression, which was marked suppressed by STAT1 knockdown (Figure 7A-B). Consistently, fludarabine, a pharmacological inhibitor of STAT1, suppressed HMGB1 expression during RLDC treatment dose-dependently (Figure 7C-D). To understand how STAT1 regulates HMGB1 expression, we first determined if the regulation occurred at transcriptional or post-transcriptional levels by analyzing mRNA. As shown in Figure 7E, *Stat1* knockdown suppressed the expression of HMGB1 mRNA during RLDC treatment, suggesting a transcriptional regulation. Bioinformatics analysis identified two potential STAT1 binding sites in the promoter region of *Hmgb1* (Figure 7F). We further assessed STAT1 binding to these two sites by chromatin immunoprecipitation (ChIP) assay. As shown in Figure 7G, after RLDC treatment, the binding of STAT1 to the predicted site 1 of *Hmgb1* promoter increased 3-4 times, but the binding to site 2 did not. Collectively, these results indicate that RLDC promotes *Hmgb1* transcription, at least in part, via STAT1.

### Upregulation of STAT1/HMGB1/NF-κB in kidney tissues of CKD patients

**Figure 8:**
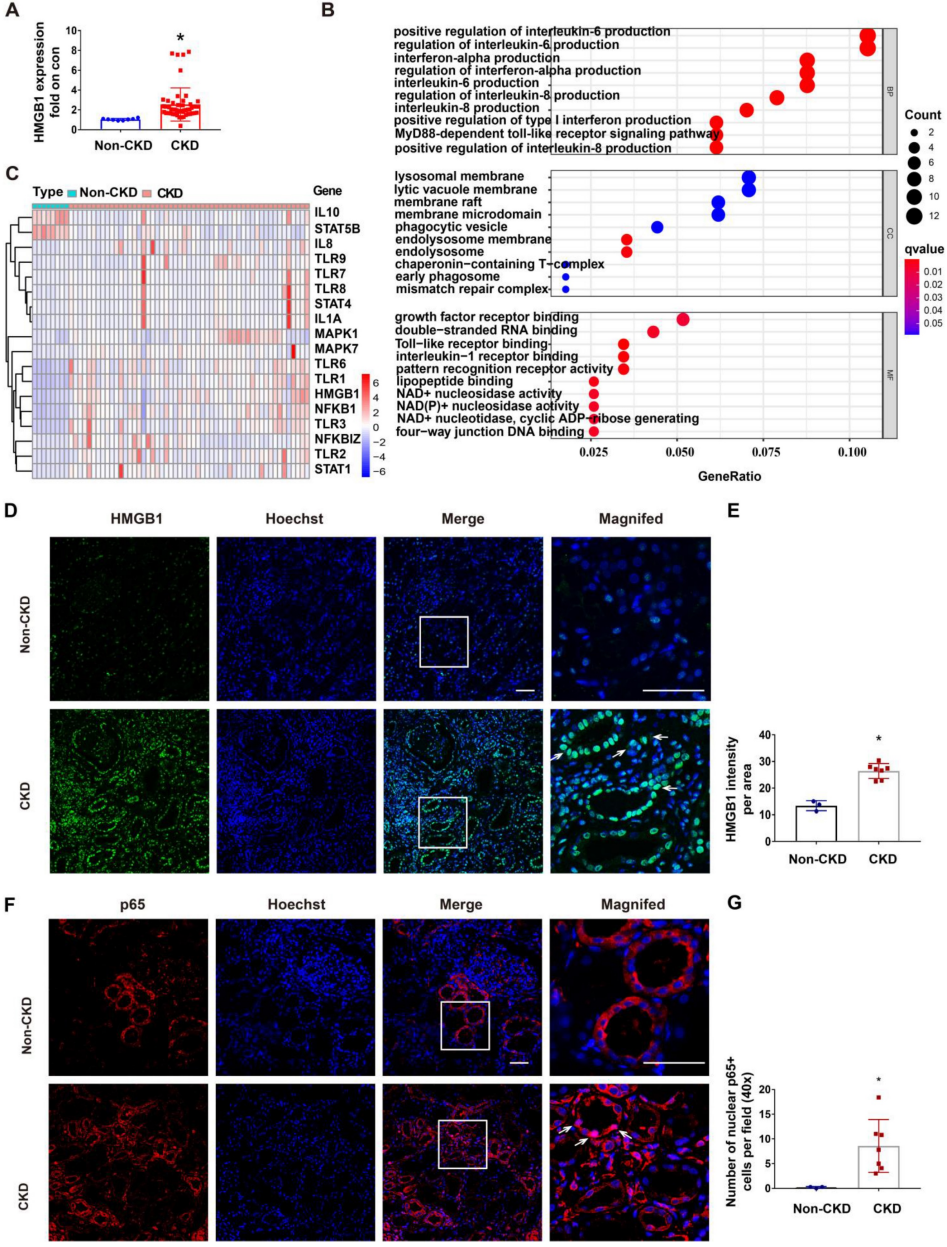
** Upregulation of STAT1/HMGB1 in kidney tissues of CKD patients.** The dataset GSE66494 deposited in GEO for kidney tissues from CKD patients and healthy donors was analyzed for gene transcription (A-C). Renal biopsies from non-CKD patient controls (n=3) and CKD patients (n=7) were assessed for the immunofluorescence staining (D-G). (A) Upregulation of HMGB1 in kidney tissues of CKD patients. Data are expressed as mean ± SEM. N: 8 healthy subjects and 54 CKD samples. * P <0.05 vs. the control group (Con). (B) Bubble graphs of the enriched gene expression pathways based on GO enrichment analysis. (C) Heat-map of differentially expressed genes (DEGs) belonging to the STAT/HMGB1 signaling pathway kidney tissues of CKD patients. (D) Representative immunofluorescence staining of HMGB1in renal biopsies from CKD patients. (E) Statistical analysis of the average fluorescence intensity of HMGB1. (F) Representative immunofluorescence staining of p65. (G) Statistical analysis of number of p65-positive cell nuclei in the area of the field. Scale bar = 50 μm. Quantitative data are expressed as mean ± SEM. N ≥ 3. * P <0.05 vs. the non-CKD group.

RLDC regimen is commonly used for cisplatin chemotherapy in cancer patients, but renal biopsy is rarely available from these patients. To determine the clinical relevance of STAT1/HMGB1 in chronic renal inflammation, we analyzed the gene transcription data from kidney tissues of CKD patients deposited in the GEO dataset GSE66494. In these CKD patients, renal expression of HMGB1 was on average 4 times higher than in control samples (Figure 8A). GO enrichment analysis on these CKD samples led to the identification of enriched gene expression pathways, which are presented in a bubble graph (Figure 8B). The most enriched biological processes (BPs), involving all up-regulated genes, were linked to inflammatory factors, including interleukin-6 production, interferon production, interleukin-8 production, and the MyD88-dependent toll-like receptor signaling pathway. Among these, the enriched molecular functions (MF) included HMGB1 signaling pathways, such as Toll-like receptor binding and pattern recognition receptor binding (Figure 8B). In addition, the majority of differentially expressed genes (DEGs) in CKD samples included STAT1, HMGB1, NF-κB, and related genes (Figure 8C). These data suggest the involvement of the STAT1/HMGB1/NF-κB pathway and associated production of inflammatory cytokines in the pathogenesis of human CKD.

To further validate the HMGB1/NF-κB axis in CKD patients, we evaluated kidney tissue samples from 3 control patients (normal nephrectomy tissues adjacent to tumors) and 7 CKD patients of various etiologies including IgA nephropathy (n = 2), and hypertension nephropathy (n = 5) (Table S2), which all had multifocal tubular atrophy (>30%) and interstitial fibrosis. Following immunofluorescence staining, we quantitated the fluorescence intensity of HMGB1 per field (40×) (Figure 8D-E) and the number of nuclear p65^+^ tubular cells per 40× field (Figure 8F-G) (n = 3). In CKD samples with multifocal tubular atrophy and interstitial fibrosis, there was a significant increase in HMGB1 expression in tubular cells and an increased rate of nuclear p65. Since a recent report showed the release of lactylated/acetylated HMGB1 from macrophages via exosomes 40, we evaluated the expression of HMGB1 by CD68+ macrophages in CKD samples with multifocal tubular atrophy and interstitial fibrosis (Figure S5). Immunofluorescence co-staining revealed an increase in infiltrated CD68^+^ cells in the renal interstitium in CKD (shown by white arrows), and a small portion of them expressed HMGB1 (shown by *). The results indicate that in addition to renal tubular cells, macrophages may also contribute to HMGB1 production in CKD kidneys.

## Discussion

Cisplatin chemotherapy leads to acute nephrotoxicity or AKI as well as chronic kidney problems in cancer patients 2, 11. While cisplatin-induced AKI has been extensively studied, much less is known about the development of chronic renal pathologies following cisplatin exposure, which, nonetheless, may involve maladaptive kidney repair characterized by tubular atrophy, chronic inflammation, and interstitial fibrosis 2, 11. In the present study, using the RLDC model of chronic cisplatin nephrotoxicity, we have demonstrated three major findings: i) RLDC induces HMGB1 activation in renal tubular cells via transcriptional up-regulation and nuclear release into the cytoplasm; ii) HMGB1 contributes to the development of chronic kidney problems after cisplatin exposure by activating NF-κB and associated production of pro-inflammatory cytokines; and iii) at the upstream, STAT1 plays a critical role in HMGB1 activation in renal tubular cells. In addition, we have verified STAT1/HMGB1 upregulation along with chronic inflammation in kidney biopsies from CKD patients. These findings suggest that targeting the STAT1/HMGB1/NF-κB signaling pathway may improve the long-term outcome after cisplatin nephrotoxicity.

The release of HMGB1 in injured kidney tubules during AKI has been suggested recently. AKI induced by renal ischemia-reperfusion 19, sepsis 25, and nephrotoxins including contrast agents 41 all causes the release of HMGB1, which may activate downstream signaling pathways for the recruitment and activation of inflammatory cells. In turn, these cells further produce inflammatory cytokines and chemokines that exacerbate kidney damage. In terms of kidney repair, HMGB1 released from pyroptotic renal tubular cells may amplify the inflammatory responses in macrophages, which contributes to renal fibrosis 42. It has also been proposed that miR-92d-3p inhibits the progression of diabetic nephropathy (DN) by inhibiting the activation of C3/HMGB1/TGF-β1 pathway and EMT 43. Conversely, Personnaz *et al.* assessed the effect of macrophage-specific *Hmgb1* gene deletion on liver, kidney, and cardiac fibrosis, and showed that macrophage-derived HMGB1 does not contribute significantly to tissue repair or fibrogenesis 44. Therefore, HMGB1 may be released from damaged kidney tubule cells to activate immune cells like macrophages for inflammation during kidney repair, although macrophage-derived HMGB1 may not play a critical role. Consistently, the latest work by Zhao et al. showed that HMGB1 in renal tubular cells promoted maladaptive kidney repair and CKD development after renal ischemia-reperfusion injury 45, although they did not examine the regulation of HMGB1.

In addition to kidney tubule cells, other cell types in the kidney may also produce and release HMGB1. For example, Yang et al. recently reported that lactylated/acetylated HMGB1 could be released from macrophages via exosome secretion, which increased the neighboring endothelium permeability 40. Consistently, we detected HMGB1 in some macrophages in renal biopsies of CKD patients. Further investigation should use cell type-specific HMGB1 knockout models to identify the cells that are responsible for HMGB1 upregulation in maladaptive kidney repair.

In our present study, HMGB1 was continuously activated and released in post-RLDC kidneys. Mechanistically, we show that upon activation, HMGB1 activated NF-κB to stimulate a robust inflammatory response. NF-κB is a transcription factor that plays important regulatory roles in inflammation, cell death, survival, proliferation, and differentiation 46, 47. In our study, blockage of HMGB1 with neutralizing antibodies or gene silencing suppressed p65/NF-κB nuclear translocation and reduced the expression of inflammatory cytokines, indicating a role of HMGB1 in NF-κB activation during maladaptive kidney repair after cisplatin nephrotoxicity. In addition to HMGB1, other factors or pathways may also contribute to NF-κB activation, such as TNF-α signaling pathway 48. Moreover, angiotensin II, a peptide hormone overproduced during kidney injury and CKD progression, has been shown to activate NF-κB 49. These pathways may work synergistically with HMGB1 to form an intertwined network for NF-κB activation and chronic inflammation in maladaptive kidney repair.

Our present study provides important insights into the mechanism of HMGB1 activation in renal tubular cells following cisplatin treatment. Interestingly, HMGB1 activation in our study includes not only the release from nuclear HMGB1 into the cytoplasm but also a remarkable upregulation of gene transcription. In immune cells, the release of nuclear HMGB1 requires the acetylation at two sites located in the nuclear localization sequence (NLS) 50. *Stat1* deletion abolished LPS- and IFN-induced HMGB1 acetylation at the NLS sites and prevented consequent release of nuclear HMGB1, demonstrating a critical role of STAT1 in HMGB1 release in these experimental conditions 23. In the present study, we show that genetic or pharmacological inhibition of STAT1 prevented the release of HMGB1 from the nucleus into the cytoplasm in renal tubular cells after RLDC treatment *in vivo* and *in vitro*, supporting a critical role of STAT1 in the release of HMGB1. Consistently, STAT1/HMGB1 was recently implicated in free light chain-induced proximal tubular injury by Upadhyay and colleagues 30.

STAT1 is known as a transcription factor that trans-activates a variety of genes in the immune response to virus and other pathogens 51. However, STAT1-mediated transcription of *Hmbg1* has not been reported. Our study has demonstrated that STAT1 may directly mediate *Hmbg1* transcription during RLDC treatment of renal tubular cells by binding to the gene promoter of *Hmbg1*. Especially, blockage of STAT1 suppressed *Hmbg1* mRNA transcription during RLDC treatment. Our bioinformatics analysis further identified two putative STAT1 binding sites in the gene promoter region of *Hmbg1* and, experimentally, our ChIP assay proved that RLDC treatment increased the binding of STAT1 to one of these sites for *Hmbg1* transcription (Figure 7). STAT1-mediated transcription of HMGB1 has an important meaning in the development of chronic kidney problems after cisplatin exposure, because this would provide a continuous source of HMGB1 that is released from renal tubules to stimulate a chronic or persistent inflammation.

Finally, our study suggests the therapeutic potential of targeting the STAT1/HMGB1/NF-κB signaling axis for the prevention of chronic kidney problems after cisplatin treatment in cancer patients. For example, inhibition of HMGB1 with a specific neutralizing antibody not only suppressed tubular damage, renal inflammation and fibrosis, but also improved kidney function (Figure 3). Of note, persistent activation of STAT proteins contributes to tumor initiation and progression, and inhibition of STAT has anti-cancer effects 52. Similarly, HMGB1 has been implicated in several types of cancers 53, 54. Therefore, inhibition of STAT1/HMGB1 may enhance cisplatin chemotherapy in tumors and protect kidneys from chronic nephrotoxicity, an intriguing possibility that requires further tests in tumor-bearing animal models.

## Supplementary Material

Supplementary figures and tables.
