# Supplementary material for: The STAT1/HMGB1/NF-κB pathway in chronic inflammation and kidney injury after cisplatin exposure
Source: Theranostics. 2023 May 8;13(9):2757–73. doi: 10.7150/thno.81406 (PMC10240827; doi:10.7150/thno.81406)
Supplement: Supplementary file 1 — Supplementary figures and tables. [file thnov13p2757s1.pdf]

Fu et al. Supplementary material

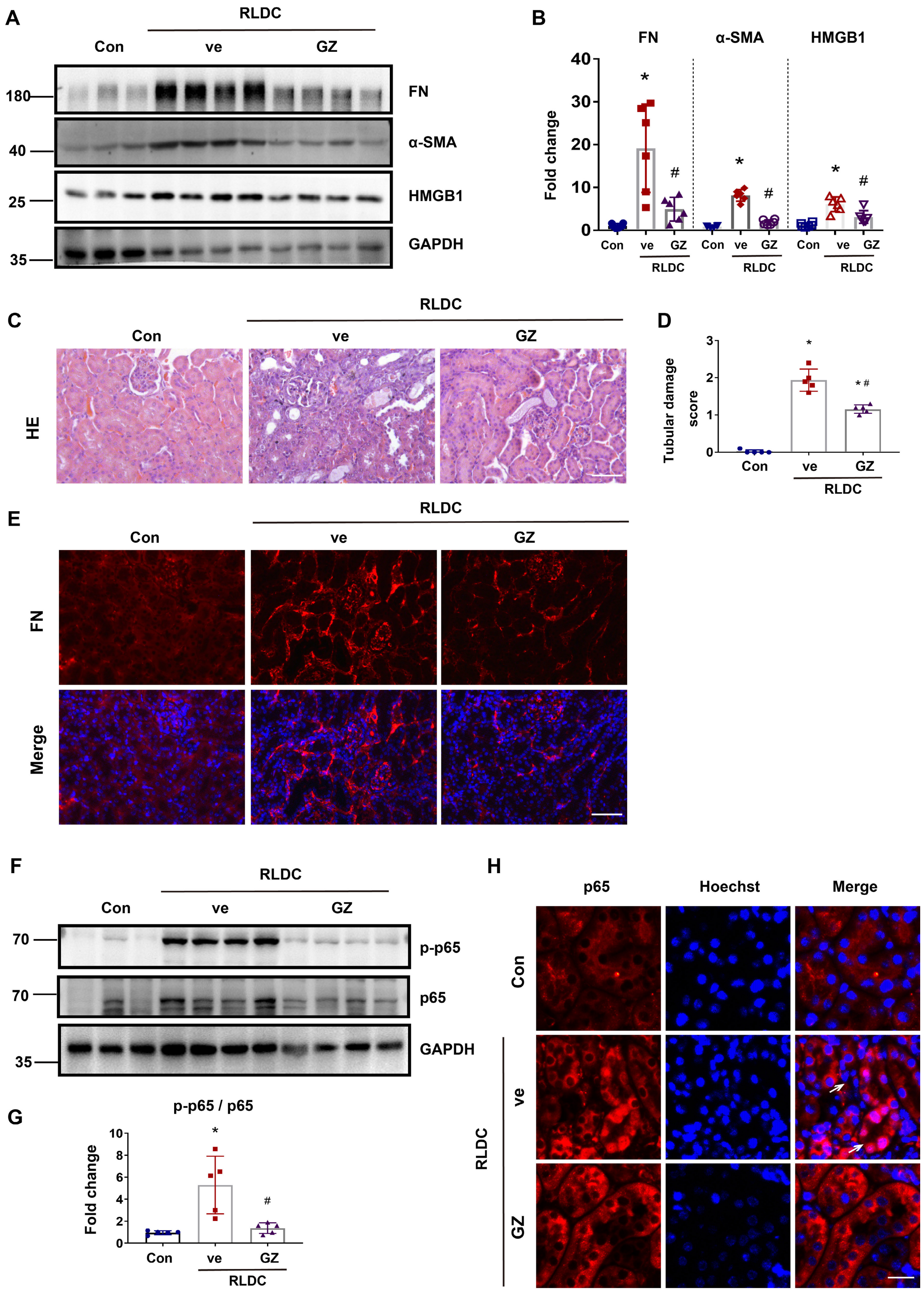

Figure S1. Effect of Glycyrrhizin (GZ), a pharmacological inhibitor of HMGB1, on kidney repair after RLDC treatment. C57BL/6 mice received a weekly injection with 8 mg/kg cisplatin for four weeks. After the last cisplatin injection, 50 mg/kg glycyrrhizin (GZ) were injected daily for one week for sample collection and analysis. (A) Representative immunoblots of FN, α-SMA and HMGB1in kidney tissues. (B) Densitometric analysis of FN, α-SMA and HMGB1 protein levels in immunoblots. (C) Representative renal histology of hematoxylin and eosin (HE)-staining showing the beneficial effect of GZ in RLDC-treated mice. Scale bar = 50 μm. (D) Kidney tubule damage score. (E) Representative images of FN immunofluorescence staining of kidney tissues. Scale bar = 50 μm. (F) Representative immunoblots of p-p65, p65 in kidney tissues. (G) Densitometric analysis of p-p65/p65 in immunoblots. (H) Representative images of immunofluorescence staining used to observe p65 entry into the nucleus. Quantitative data are expressed as mean ± SEM. N = 5. * P <0.05 vs. the untreated control group (Con); # P < 0.05 vs. (RLDC+ve) group.

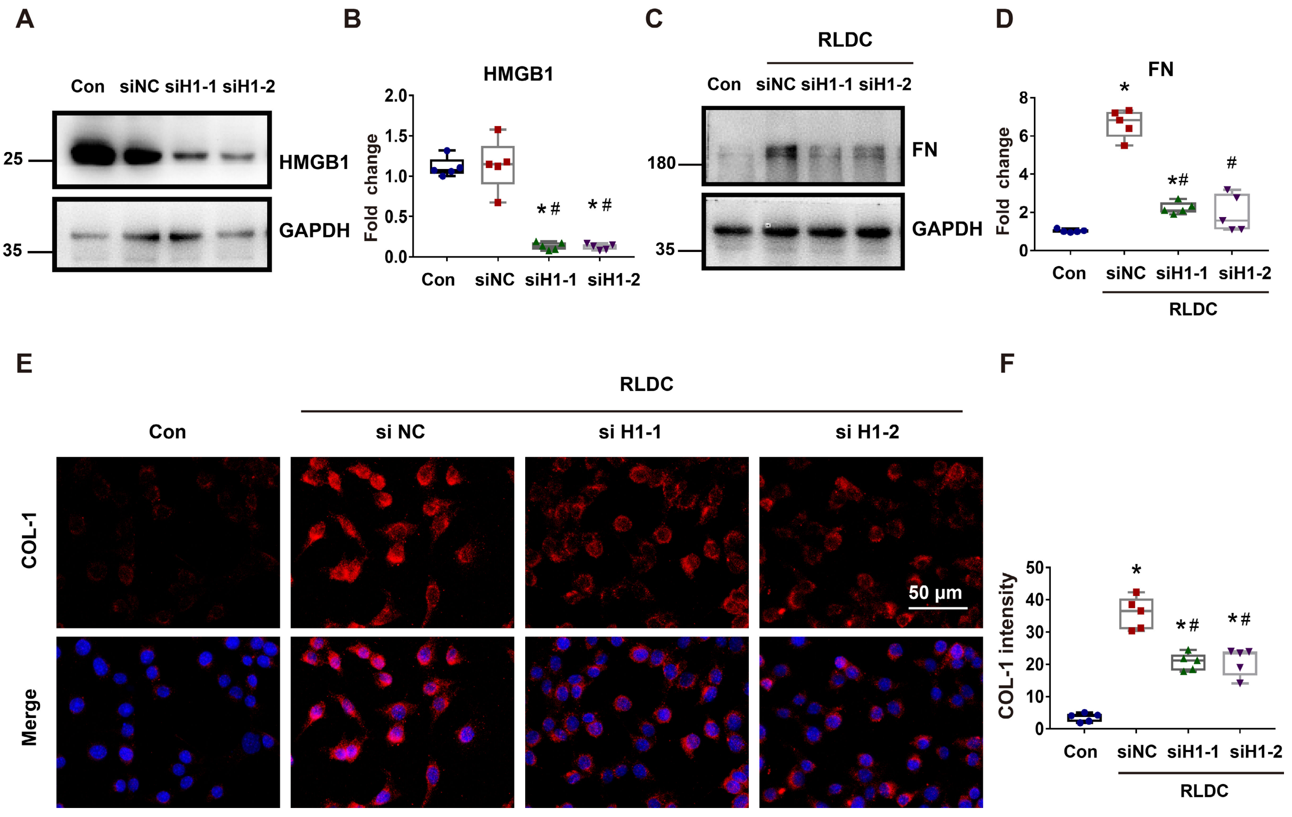

Figure S2. ***Hmgb1* knockdown reduces RLDC–induced fibrotic phenotype in renal tubular cells.** BUMPT cells were transfected with 50 nM of HMGB1 siRNA1 (siH1-1), HMGB1 siRNA2 (siH1-2), or a negative control siRNA (siNC), and then subjected to 4 days of RLDC treatment or

control incubation (Con). (A) Representative immunoblots of HMGB1 and GAPDH (loading control). (B) Densitometry of HMGB1 in immunoblots. (C) Representative immunoblots of FN and GAPDH. (D) Densitometry of FN in immunoblots. (E) Immunofluorescence analysis of COL-1 (red) in BUMPT cells with Hoechst staining of their nuclei (blue). Scale bar = 50 µm. (F) Quantitative analysis of COL-1 staining. Quantitative data are expressed as mean ± SEM. N = 5. * $P < 0.05$ vs. the control group without RLDC treatment (Con). # $P < 0.05$ vs. RLDC/siNC group.

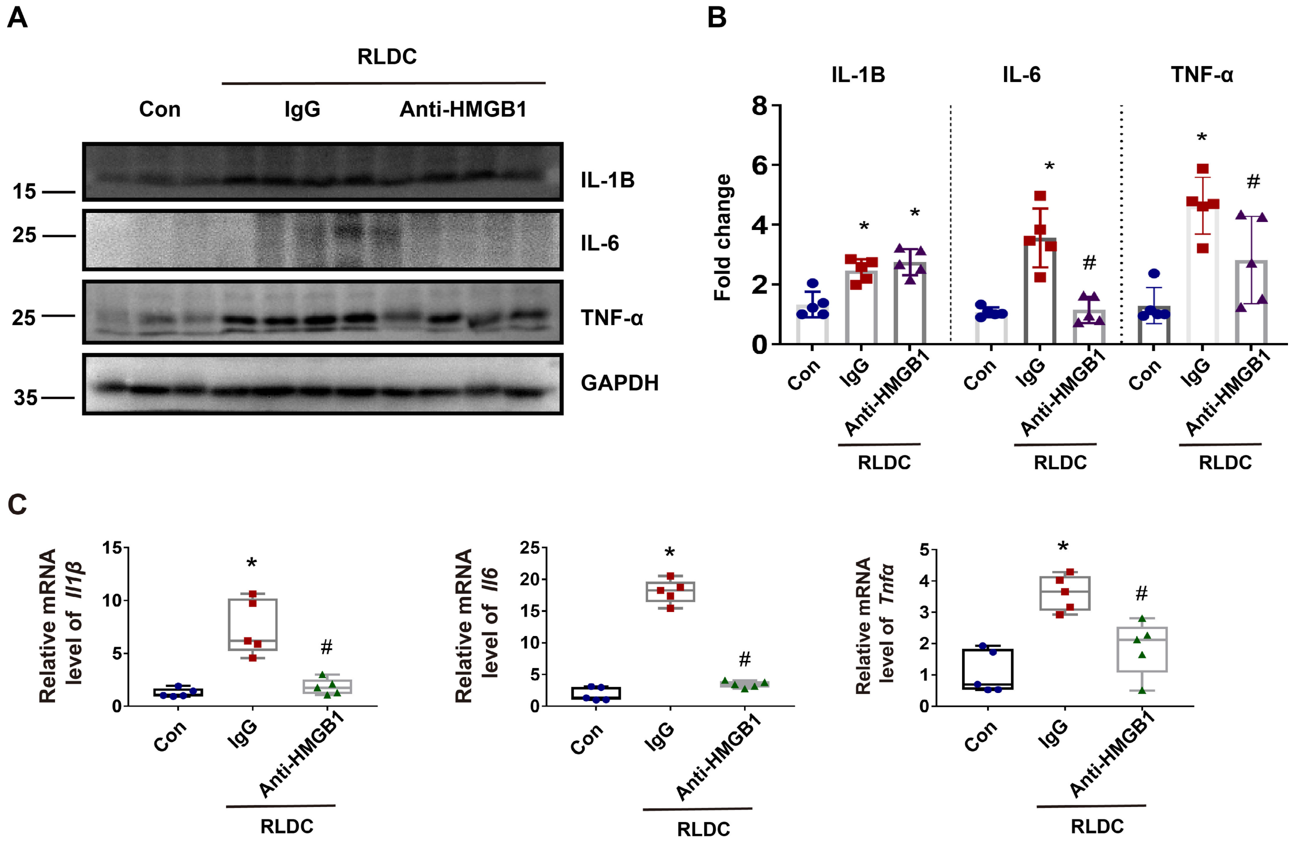

Figure S3. **Anti-HMGB1 neutralizing antibody suppresses *Il-1β*, *Il-6* and *Tnf-α* expression in post-RLDC mouse kidneys.** Male C57BL/6 mice received a weekly injection of 8 mg/kg cisplatin for four weeks. After the last injection, anti-HMGB1 antibody (Anti-HMGB1) or IgG isotype control (IgG) were injected daily for one week until sample collection. (A) Representative immunoblots of IL-1β, IL-6, TNF-α and GAPDH (loading control). (B) Densitometry of IL-1β, IL-6, TNF-α in immunoblots. (C) Relative quantification of the levels of *Il-1β*, *Il-6*, and *Tnf-α* mRNAs by qRT-PCR. The expression of the target genes was normalized to GAPDH mRNA and expressed as fold change compared to control kidneys (Con). N = 5. *P<0.05 vs. the control group (Con), #P < 0.05 vs. RLDC/control isotype-treated group.

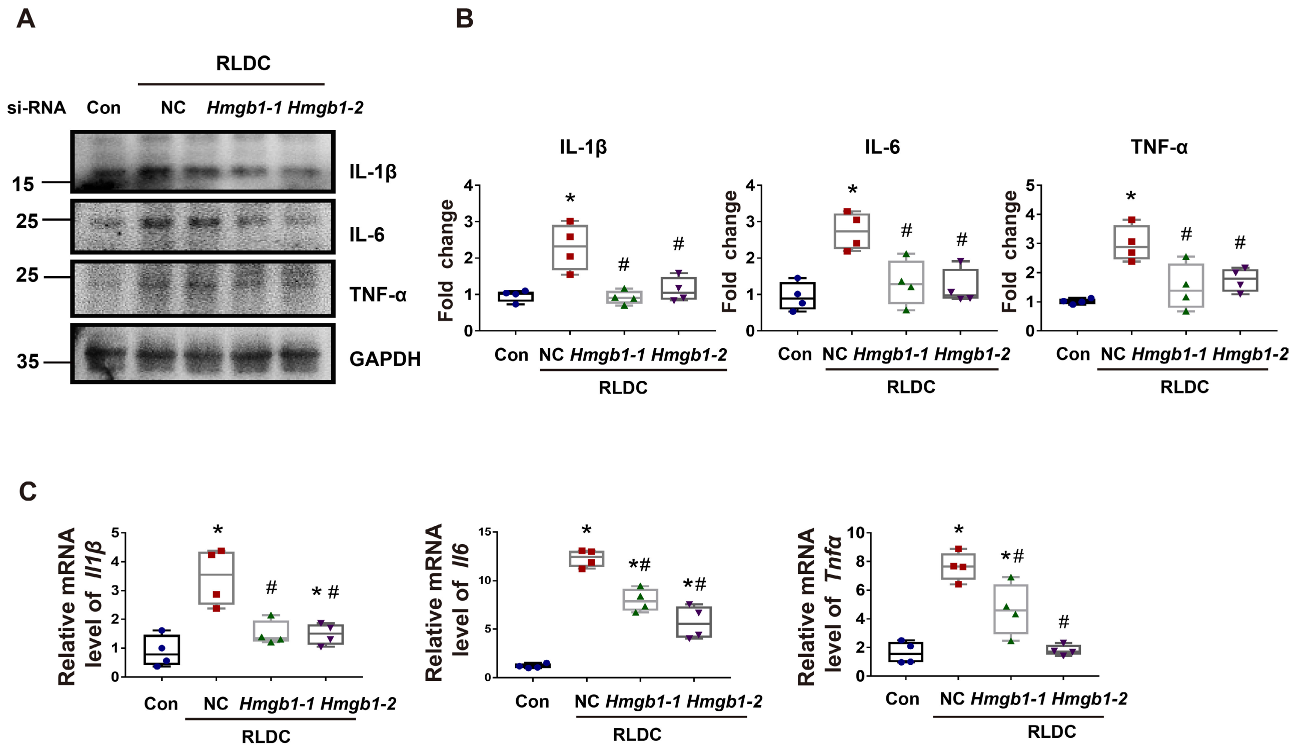

Figure S4. ***Hmgb1*** **knockdown decreases pro-inflammatory cytokines in RLDC-treated renal tubular cells.** BUMPT cells were transfected with 50 nM *Hmgb1* siRNA (siH1-1, siH1-2), or negative control siRNA (siNC), and then subjected to 4-day basic RLDC treatment. Control cells were left untreated (Con). (A) Representative immunoblots detecting IL-1β, IL-6, TNF-α, and GAPDH. (B) Quantitative analysis of IL-1β, IL-6, and TNF-α immunoblots. (C) Relative quantification of the levels of *Il-1β*, *Il-6*, and *Tnf-α* mRNAs by qRT-PCR. The expression of the target genes was normalized to GAPDH mRNA and expressed as fold change compared to control cultures (Con). Data are expressed as mean ± SEM. N = 4. *P<0.05 vs. the untreated cultures (Con); #P < 0.05 vs. RLDC/control siRNA-treated cultures.

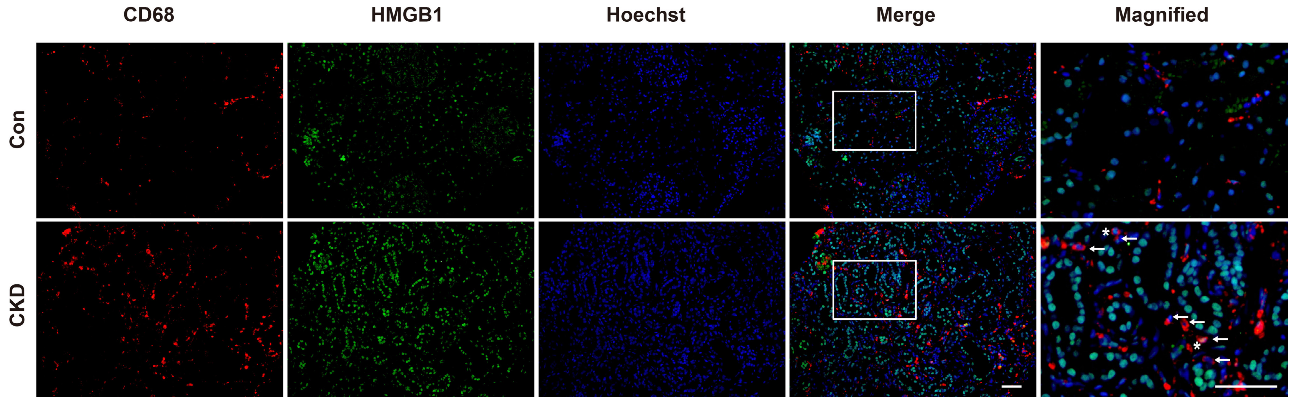

Figure S5. Immunofluorescence co-staining of HMGB1 with CD68 in renal biopsies of CKD patients. To further validate the expression of HMGB1 produced by macrophages in CKD patients, we performed immunofluorescence co-staining of CD68 and HMGB1 using tissue samples from 3

control patients (normal kidney tissue adjacent to cancer) and 7 CKD patients with different etiologies, including IgA nephropathy (n = 2), and hypertensive nephropathy (n = 5). In CKD with multifocal tubular atrophy and interstitial fibrosis, HMGB1 expression was significantly increased in renal tubular cells and in some CD68+ macrophages. Arrows refers to CD68+ cells in the interstitium, and * refers to CD68+HMGB1+ cells. Scale bar = 50 μm.

Table S1. Primer sequences used for quantitative RT-PCR.

Gene	Forward (5′-3′)	Reverse (5′-3′)
Il-1β	GAAATGCCACCTTTTGACAGTG	CTGGATGCTCTCATCAGGACA
Il-6	TCCAGTTGCCTTCTTGGGAC	GTACTCCAGAAGACCAGAGG
Tnf-α	CAGGCGGTGCCTATGTCTC	CGATCACCCCGAAGTTCAGTAG
Hmgb1	GGGAGGAGCACAAGAAGAAGCA	GGGCGGTACTCAGAACAGAACAAG
Tlr2	GACGCTGGAGGTGTTGGATGTTAG	AAAGTGGTTGTCGCCTGCTTCC
Tlr4	CCGCTTTCACCTCTGCCTTCAC	TGCCGTTTCTTGTTCTTCCTCTGC
Myd88	CGCCGCCTATCGCTGTTCTTG	TGCCTCCCAGTTCCTTTGTTTGTG
Gapdh	AGGTCGGTGTGAACGGATTTG	GGGGTCGTTGATGGCAACA

Table S2. Demographic and clinical information of the CKD patients.

**Number**	**Age**	**Gender**	**CKD staging**	**eGFR(ml/min/1.73m^2)**	**Diagnosis**	**Tubular atrophy (%)**	**Interstitial fibrosis (%)**
1	52	M	IIIa	48.7	IgA nephropathy	70	70
2	36	F	IIIa	45.5	IgA nephropathy	30	30
3	64	M	IIIb	32.3	Hypertensive nephropathy	30	30
4	25	M	IIIa	46.1	Hypertensive nephropathy	70	70
5	59	M	IV	16.7	Hypertensive nephropathy	30	30
6	55	M	II	72.3	Hypertensive nephropathy	30	30
7	38	M	IV	19	Hypertensive nephropathy	70	70
